# Glucanase Induces Filamentation of the Fungal Pathogen *Candida albicans*

**DOI:** 10.1371/journal.pone.0063736

**Published:** 2013-05-30

**Authors:** Hongbin Xu, Clarissa J. Nobile, Anna Dongari-Bagtzoglou

**Affiliations:** 1 Department of Oral Health and Diagnostic Sciences, University of Connecticut Health Center, Farmington, Connecticut, United States of America; 2 Department of Microbiology and Immunology, University of California San Francisco, San Francisco, California, United States of America; University of Wisconsin Medical School, United States of America

## Abstract

*Candida albicans* is the most common human fungal pathogen. Many organisms, including *C. albicans*, secrete glucanases under different environmental conditions. Here, we report a novel role for *beta*-1, 3- glucanase in inducing *Candida albicans* to form filaments at 22°C and enhancing filamentation at 37°C in nutrient-rich medium. Quorum sensing, the *efg1*-signaling and *cek1* MAP kinase pathways are involved in this process. Our data suggest that the natural antifungal agent *beta*–glucanase may support morphologic transformation of *Candida albicans* at a wide range of ambient temperatures.

## Introduction

*Candida albicans* is a fungus that can undergo multiple morphological transitions. *C. albicans* is known to infect a wide variety of hosts from warm-blooded animals to insects [1]. Hyphal formation is essential for virulence and invasion in a variety of hosts [2]. Many environmental stimuli can stimulate *Candida albicans* to form hyphae, including, but not limited to, serum, CO_2_, quorum sensing molecules, and pH [3]–[6]. However, most of these stimuli require incubation physiological temperature (37°C) for efficient hyphal growth. Although hyphal formation has been reported at lower temperatures, the conditions used were complex, requiring a matrix [7] or amino acid-containing liquid synthetic medium [8].

Many organisms have *beta*-1, 3-glucanase genes and secrete glucanases into the environment, including *C. albicans* [9], [10]. *C. albicans* possesses three cell wall related exo-beta-1, 3-glucanases, Xog1, Exg2, and Spr1 [11]. CAMP65 was also identified as a putative *beta*-glucanase and is required for hyphal morphogenesis [12]. Although these hydrolytic enzymes are believed to play cell wall remodeling roles during growth and morphogenesis in filamentous fungi, they may play multiple physiological roles, which have not been fully elucidated in *C. albicans*. In this work, we show that *beta*-1, 3- glucanase, an enzyme produced by many bacteria, fungi and plants [13]–[15], permits *C. albicans* to overcome the temperature requirement for hyphal transformation in yeast peptone dextrose (YPD) medium.

## Materials and Methods

### Candida albicans strains and growth conditions

The wild type strain SC5314, and strains SN425 (reference stain), SGH284 (a biosensor reporter strain), HLC54 (*egf1/cph1 double mutant*), *cph1* mutant, *cek1* mutant, CJN2302 (*efg1* mutant), *cht2* mutant and CJN2318 (*efg1* revertant) were grown in YPD medium and maintained on YPD agar plates (Table 1). These strains were described in detail elsewhere [2], [16]–[18]. To induce filamentation, the cells were grown to stationary phase overnight in YPD broth, and then inoculated in 5 ml glucanase-supplemented media (YPD, DMEM or RPMI) at 10^6^ cells per ml and incubated at 22°C, 30°C or 37°C, for 18 h without shaking. For glucanase dose response experiments *beta*-1, 3- glucanase/lyticase, purified from two microbial sources (from *Arthrobacter luteus* or *Trichoderma harzianum,* Sigma), was added in YPD at concentrations ranging between 0.1–100 µg/ml and organisms were inoculated and incubated as described above. Glucanase inactivated by heating at 95°C for 10 minutes was used as control. To induce true hyphae, 10% FBS was used as positive control and organisms were grown at 37°C in an aerobic incubator with 5% CO_2_. Hyphal units were enumerated under phase contrast microscopy.

**Table 1:** 

Strains	Genotype	Reference
SC5314	Wild type, clinic isolate	[37]
SGH284	ura3Δ::λimm434 ARG4 his1::hisG::pHIS1-pTDH3-GFP-tADH1 HWP1::pHWP1-RFP-tADH1-URA3, ura3Δ:: λimm434 arg4::hisG his1::hisG HWP1	[26]
CJN2302	ura3Δ:: λimm434::URA3-IRO1 arg4::hisG his1::hisG leu2::hisG::CdARG4 efg1Δ::CmLEU2, ura3Δ:: λimm434 arg4::hisG his1::hisG leu2::hisG efg1Δ::CdHIS1	[16]
CJN2318	ura3Δ:: λimm434::URA3-IRO1 arg4::hisG his1::hisG leu2::hisG::EFG1-CdARG4, efg1Δ::CmLEU2, ura3Δ:: λimm434 arg4::hisG his1::hisG leu2::hisG efg1Δ::CdHIS1	[16]
SN425	ura3Δ:: λimm434::URA3-IRO1 arg4::hisG::CdARG4 his1::hisG leu2::hisG::CdHIS1, ura3Δ:: λimm434 arg4::hisG his1::hisG leu2::hisG::CmLEU2	[16]
HLC54	ura3::1 imm434/ura3::1 imm434 cph1::hisG/cph1::hisG efg1::hisG/efg1::hisG-URA3 hisG	[2]
*cph1* mutant	his1Δ/his1Δ, leu2Δ/leu2Δ, arg4Δ/arg4Δ, URA3/ura3Δ::imm434,IRO1/iro1Δ::imm434, orf19.4433Δ::C.dubliniensisHIS1/ orf19.4433Δ::C.maltosaLEU2	[17]
*cek1* mutant	his1Δ/his1Δ, leu2Δ/leu2Δ, arg4Δ/arg4Δ, URA3/ura3Δ::imm434,IRO1/ iro1Δ::imm434, orf19.2886Δ::C.dubliniensisHIS1/orf19.2886Δ::C.maltosaLEU2	[17]
*cht2* mutant	ura3Δ:: λimm434 arg4::hisG his1::hisG, cht2::Tn7-UAU1 ura3Δ:: λimm434 arg4::hisG his1::hisG cht2::Tn7-URA3	[18]

### Fluorescence Microscopy

For staining the cell wall or nuclear material, the cells were collected by centrifugation, washed with PBS and stained with Calcofluor white (Sigma) or Hoechst 33258 (BIO-RAD), respectively, for 10 minutes. After washing in PBS the cells were mounted on slides and observed under a fluorescence microscope. To examine the effect of glucanase treatment on the cell wall glucan, cells were stained with a monoclonal antibody highly specific for (1→6) branched, (1→3)-β-D-glucans (BFDiv, Biothera), as we previously described [19]. To examine cell viability, a fluorescence LIVE-DEAD viability stain kit (Molecular Probes) was used to stain the cells, according to manufacturer's instructions. In some experiments farnesol or tyrosol were added to the media at 10–200 µM (Sigma) [20], [21].

### Quantitative Real- time PCR Assay

The assay was described in detail previously [22]. Briefly, 5 ml cell cultures were grown as described above and total RNA was extracted using the RiboPure yeast kit (Ambion, Inc.). RNA concentrations and quality were determined by measuring the absorbance at 260 nm and 280 nm (ND-1000 spectrophotometer, NanoDrop Technologies). Equal amounts of RNA (3 μg in 20 μl reactions) were reverse transcribed with oligo(dT) primers using Superscript reverse transcriptase II (Invitrogen). Primers used were as follows: EFB1, Forward: 5′- CAT TGA TGG TAC TAC TGC CAC -3′; Reverse: 5′- TTT ACC GGC TGG CAA GTC TT -3′. HWP1, Forward: 5′- TGG TCC AGG TGC TTC TTC TT -3′; Reverse: 5′- GGT TGC ATG AGT GGA ACT GA-3′. ALS3, Forward: 5′- CCA CTT CAC AAT CCC CAT C -3′; Reverse: 5′- CAG CAG TAG TAA CAG TAG TTT CAT C -3′. HYR1, Forward: 5′- CGT CAA CCT GAC TGT TAC ATC -3′; Reverse: 5′- TCT ACG GTG GTA TGT GGA AC -3′.UME6, Forward: 5′- CAG TGG TAA TGG CAC TAA CAC C -3′; Reverse: 5′- GCA CAA CCT CCA CAA ATT GGT G -3′. CHT2, Forward: 5′- CAA ACC ACT TCC TAC CCT GTTG- 3′; Reverse: 5′-GAT GTT GGG TAT GTA ACT GGGG -3′. CHT3, Forward: 5′- CAA CTT CGT CGA CAA GTT TATC -3′; Reverse: 5′-AGA AGA TGG TGT AAC AAC TGGG -3′. EFB1 gene was used as internal control to normalize the gene expression level [22]. Real time PCR was performed with BIO-RAD CFX96 cycler and iQ™ SYBR® Green Supermix kit (BIO-RAD) was used to set up all reactions according to the manual.

## Results and Discussion

**Figure 1:**
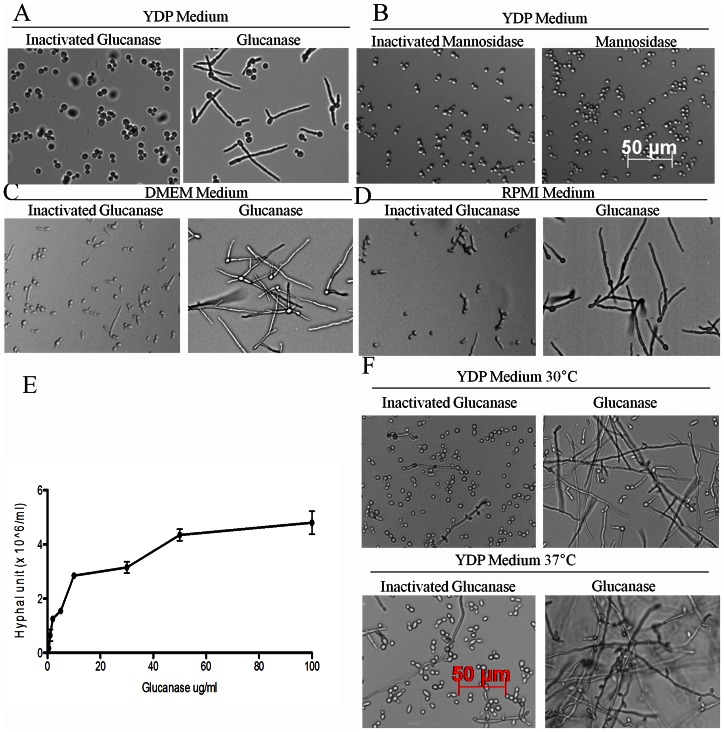
Filamentation induced by glucanase. A: Photomicrograph of hyphae induced by glucanase (100 µg/ml). The cells were grown in 5 ml YPD medium in BD 14 ml plastic tubes at 22°C without shaking for 18 h. Heat-inactivated glucanase was used as control at the same concentration. B: Mannosidase (100 µg/ml) did not induce hyphae under the same conditions as glucanase. Heat- inactivated mannosidase was used as control at the same concentration. C, D: Glucanase (100 µg/ml) induced all planktonic cells to grow as filaments in DMEM or RPMI media. Cells were incubated in respective media at 22°C without shaking for 18 h. Heat inactivated glucanase was used as control at the same concentration. E: Hypha formation was dependent on glucanase concentration in YPD medium. Cells were inoculated in YPD media containing 0.1–100 µg/ml of glucanase and incubated at 22°C without shaking for 18 h. F, G: Glucanase (100 µg/ml) enhanced filamentation at 30°C and 37°C in YPD medium. Cells were incubated in a water bath without shaking for 18 h. Filaments were longer than those at 22°C.

*C. albicans* standard laboratory strain SC5314 grows as a yeast form in YPD at 22°C [6], [23]. In order to test the effect of glucanase under these conditions, we inoculated 10^6^ cells per ml in 5 ml YPD medium containing 50 µg/ml β-1, 3-glucanase. After overnight growth at 22°C without shaking, we found that glucanase induces *C. albicans* to form long filaments (Fig. 1.*A*). *C. albicans* SC5314 does not form filaments in the presence of heat-inactivated glucanase under these conditions (Fig. 1.*A*). Results with β-1, 3-glucanase from two different microbial sources were similar (data not shown). To confirm that this effect was specific to glucanase, we tested mannosidase under the same conditions and found that it did not induce filamentation (Fig. 1.*B*). In glucanase –supplemented DMEM or RPMI media without serum, filamentation was even more pronounced, with almost 100% of cells forming filaments (Fig. 1.*C* and *D*). Glucanase showed a dose-dependent effect on filamentation with an active concentration as low as 0.5 µg/ml, whereas concentrations above 50 µg/ml did not increase the number of hyphal cells further (Fig. 1.*E*). Finally, to test whether filamentation in the presence of glucanase was temperature-dependent, we examined the effect of glucanase at 30°C and 37°C. Filamentation was increased by glucanase significantly at these temperatures, but in contrast to 22°C where *C. albicans* cannot form filaments in YPD without glucanase, a lower degree of filamentation is possible at these higher temperatures in YPD medium in the absence of glucanase (Fig. 1.F and G).

**Figure 2:**
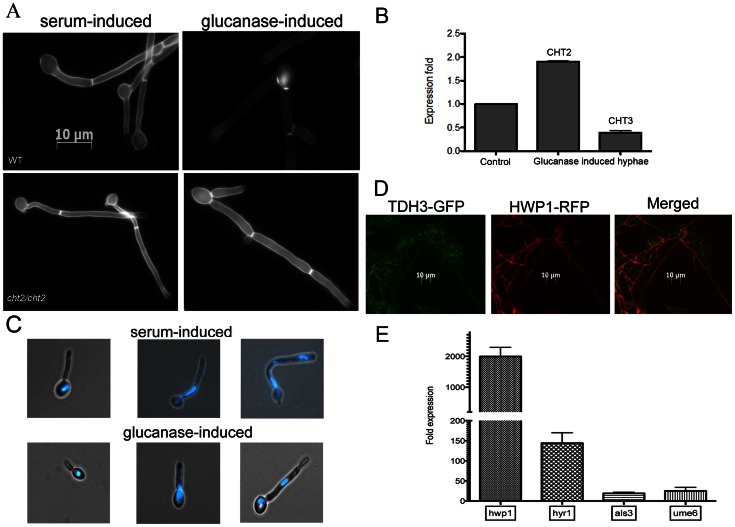
Microscopic examination of glucanase-induced hyphae. A: Calcofluor white stain of serum –induced (10% FBS at 37°C) and glucanase-induced hyphae (YPD 22°C) of wild type organisms and *cht2* mutant. Glucanase-induced filaments of the wild type organisms did not stain as brightly with CFW compared to serum-induced hyphae. In addition, the first septum was located at the mother-bud neck, whereas in serum-induced hyphae, the first septum was located within the germ tube. In the *cht2* mutant, glucanase-induced filaments had a similar septal localization but the intensity of the CFW staining was indistinguishable from serum-induced hyphae (lower panel). B: Real-time RT-PCR of the CHT2 and CHT3 genes in glucanase-treated SC5314 cells. CHT2 gene expression was significantly increased in glucanase-induced hyphae (p<0.05). C: Hoechst 33258 DNA dye showing nuclear divisions of serum –induced and glucanase-induced filaments. Mitosis of serum –induced germ tubes took place within the tubes but mitosis of glucanase-induced hyphae was at the neck between mother cells and germ tubes. D: The biosensor reporter strain SGH284 was exposed to 100 µg/ml glucanase for 18 h at 22°C without shaking. GFP (green) is expressed both by yeast and hyphae under these conditions, since it is driven by a constitutive promoter (*TDH3*-*GFP*). In contrast, only filaments express RFP (red) driven by the *HWP1* promoter (*HWP1-RFP*), indicating that glucanase induces a hyphal response. E: Real-time RT-PCR of hypha-specific genes showed that *HWP1* gene expression was increased more than 2000 fold, *HYR1* more than 100 fold, *ALS3* more than 20 fold and *UME6* more than 40 fold, compared to controls treated by inactivated glucanase.

We found that glucanase treatment does not reduce the immunoreactivity of the filaments to an anti-β-glucan antibody, arguing against the possibility of major hydrolytic dissolution of the β-glucan layer of the cell wall under these conditions (not shown). However, we noticed that glucanase treatment reduces binding of Calcofluor White (CFW), a chitin-specific dye, to the cell wall (Fig2. *A*). This suggested that glucanase treatment reduced the cell wall chitin content. We hypothesized that glucanase may upregulate chitinase gene expression, which may in turn lead to chitin degradation, and analyzed the expression of the CHT2 and CHT3 genes. These chitinase genes are repressed during hyphal morphogenesis as they play roles in cell separation [24], [25]. In agreement with this, CHT3 gene transcription was downregulated during exposure to glucanase (Fig. 2.*B*). However, CHT2 gene transcription was significantly increased, suggesting that this enzyme may be responsible for the reduction in the chitin signal in CFW-stained filaments (Fig. 2.*B*). To confirm the role of CHT2 in this process we stained a *cht2* mutant with CFW and found that the intensity of CFW binding on the glucanase-induced filaments was restored to levels similar to serum-induced hyphae in the mutant or the wild type background (Fig. 2.*A*). Collectively these data support the notion that glucanase reduces the chitin content in the cell wall of *C. albicans* by upregulating CHT2 gene expression.

CFW staining also showed that the sides of the cell walls in glucanase-induced filaments were parallel, resembling true hyphae. However, we noticed that the first septum was located at the mother-bud neck, whereas in 10% serum-induced hyphae, the first septum was located within hyphae (Fig. 2.*A*). The septal localization pattern was medium-independent (not shown) and suggests that in glucanase-induced hyphae, the nuclear division occurred across the mother-bud neck, as seen in yeast or pseudohyphae [4]. To examine nuclear division, we stained the DNA with Hoechst stain, a DNA-specific dye. As shown in Fig. 2.*C*, in 10% serum- induced hyphae, nuclear division took place throughout the filament, but in glucanase- induced hyphae, nuclear division was noted across the mother-bud neck, as is typical of yeast cells.

In order to test whether hyphal specific genes were expressed in glucanase-treated cultures, we used a dual approach. First the biosensor reporter strain SGH284 was used which has a hyphal-specific *HWP1-RFP* reporter, and a constitutive *TDH3-GFP* reporter that expresses GFP in both yeast and hyphal forms [26]. As shown in Fig. 2.*D*, the RFP signal could be observed on the surface of the glucanase-induced filaments but not on yeast cells, showing that the hypha-specific *HWP1* gene was strongly expressed. To confirm this finding we analyzed *HWP1* gene expression by qRT-PCR and included other genes that are strongly upregulated in hyphae-inducing conditions such as *HYR1*, *ALS3* and *UME6* [27]-[29]. Indeed, a strong upregulation of all four hyphal-associated genes was observed, with *HWP1* gene expression level increasing about 2,000 fold, *HYR1* about 150 fold, *ALS3* about 19 fold, and *UME6* about 25 fold, compared to inactivated glucanase controls (Fig. 2.*E*).

**Figure 3:**
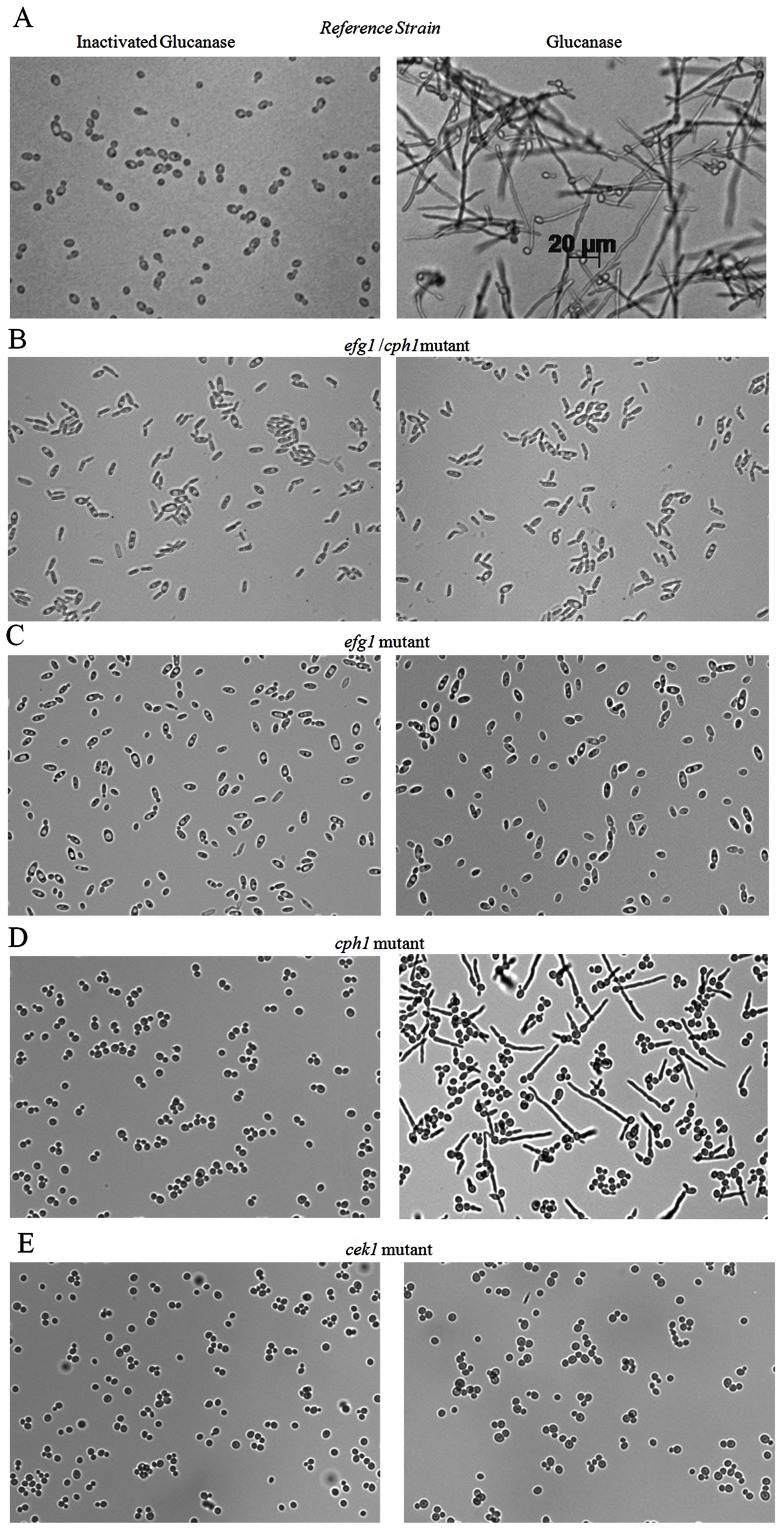
Hypha formation was absent in the efg1/cph1 double mutant, efg1 mutant and cek1 mutant. Effect of glucanase (100 µg/ml) or inactivated glucanase on filamentation of reference strain (A), *efg1/cph1* double mutant (B), *efg1* single mutant (C), *cph1* single mutant (D) and *cek1* single mutant (E). Only the *cph1* single mutant formed filaments when exposed to glucanase (D).

We then investigated the signaling pathway involved in glucanase-induced filament formation. Hyphal development in *C. albicans* is under transcriptional control of two principal signaling pathways, the MAP kinase pathway, predominantly represented by the transcription factor *CPH1*, and the cAMP pathway, represented by *EFG1*. The *efg1/cph1* double mutant is unable to respond to most hyphae-inducing environmental stimuli; we thus first examined the effects of glucanase on this double mutant strain [2]. As expected the double mutant did not form hyphae in response to glucanase (Fig. 3.A). We next tested *efg1* and *cph1* single mutants for their response to glucanase in order to further dissect which of the two signaling pathways is involved. As shown in Fig. 3.C, the *efg1* mutant did not form hyphae in contrast to the reference (Fig. 3.A) and complemented strains (not shown), suggesting that the effects of glucanase on filamentation are under the control of the cAMP signaling pathway. In contrast, the *cph1* mutant formed hyphae in the presence of glucanase (Fig. 3.D), confirming that this transcription factor is not involved in the filamentation response. Cek1 is a MAP kinase that acts in concert with Efg1 in the adaptation response to defective cell wall glycosylation [30], [31]. To examine the involvement of this MAP kinase in the glucanase-induced filamentation response, we tested the ability of a *cek1* mutant to form filaments under these conditions. As seen in Fig. 3.E, the *cek1* mutant did not form filaments in response to glucanase, indicating that Cek1is involved in glucanase-induced filamentation. These results also suggest that Cek1 is involved in phosphorylation of Efg1, or that there is cross-talk between the *EFG1* and *CEK1*/MAP kinase signaling pathways during adaptation to glucanase exposure.

**Figure 4:**
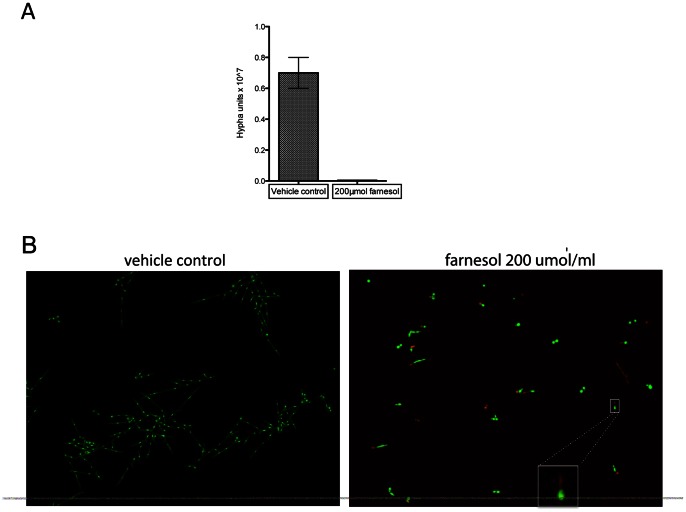
Farnesol effect on the yeast-hyphae transition induced by glucanase in YPD at 22°C. A: Farnesol (200 μmol) inhibits filamentation induced by glucanase (100 μg/ml). Methanol was used as vehicle control. Methanol did not affect *C. albicans* filamentation under this condition. B: Live-Dead cell viability staining assay. Live cells were stained with SYTO9 (green) and dead cells were stained with propidium iodide (red). Staining shows that hyphal inhibition of farnesol (200 μmol) presence of glucanase (100 μg/ml) is mediated by germ tube death (red). Methanol vehicle control did not affect the *C. albicans* viability.

In *C. albicans*, cell-density dependent quorum sensing molecules can affect the transition of yeast to hyphal forms and vice versa [20], [21]. In order to test whether glucanase- induced filamentation is dependent on cell density, we inoculated different yeast cell concentrations and treated them with 100 µg/ml glucanase. We found that the effect of glucanase was dependent on the initial inoculum size, since filamentation was not observed when the inoculum exceeded 10^6^cells/ml (data not shown). We thus hypothesized that quorum sensing molecules can limit the effect of glucanase on filamentation, and tested the effect of farnesol, a known quorum sensing molecule that inhibits hyphal formation [21]. Farnesol inhibited glucanase-induced filamentation (Fig. 4*A*), although a few germ tubes were still present that appeared to be dead as seen with the Live-Dead dye (Fig. 4*B*). We also tested another quorum sensing molecule, tyrosol [20] that supports hyphal formation, but found no enhancement on the hyphae-inducing effect of glucanase (data not shown).

*C. albicans* is often found along with other microorganisms in a microbial community called a biofilm [19], [32]. *C. albicans* and many other organisms possess glucanase genes and secrete glucanases into the environment that can digest glucan. Cell wall associated glucanases in *C. albicans* remodel cell wall during cell division and morphogenesis [12], [33]. In the plant kingdom, numerous plants also secrete glucanases, which may act as anti-fungal agents that lyse the fungal cell wall and protect them from fungal invasion [34]-[36]. Here, we showed evidence that *beta*-1, 3-glucanases can induce filamentation of *C. albicans*, even at low temperatures and that this process is controlled by the efg1 and cek1 pathways. To our knowledge, this is the first report that *beta*-1, 3-glucanases from bacteria or fungi induce filamentation or any physiological response in *C. albicans*, and our findings suggest that this may be a protective adaptive response to these cell wall glucan-damaging enzymes. The glucanase-exposed yeast cells grow pseudohyphae first, but then continue to develop into true hyphae, as suggested by expression of hyphae-enriched genes that are associated with virulence in mammalian hosts. These observations also suggest that the presence of glucanases in polymicrobial biofilms may influence the virulence potential of *C. albicans* by increasing filamentation in a variety of host environments.
